# Supplementary material for: Multimodal assessment of non-alcoholic fatty liver disease with transmission-reflection optoacoustic ultrasound
Source: Theranostics. 2023 Jul 24;13(12):4217–28. doi: 10.7150/thno.78548 (PMC10405839; doi:10.7150/thno.78548)
Supplement: Supplementary file 1 — Supplementary figures. [file thnov13p4217s1.pdf]

SUPPLEMENTARY MATERIALS FOR:

Multimodal assessment of non-alcoholic fatty liver disease with transmission-reflection optoacoustic ultrasound

Berkan Lafci[1,2], Anna Hadjihambi[3,4,5], Madita Determann[6], Christos Konstantinou[3,4,5], Clara Freijo[7], Joaquin L. Herraiz[7,8], Sena Blümel[6], Luc Pellerin[5,9], Neal C. Burton[10], Xosé Luís Deán-Ben[1,2], and Daniel Razansky[1,2,*]

[1] Institute of Pharmacology and Toxicology and Institute for Biomedical Engineering, Faculty of Medicine, University of Zurich, Switzerland

[2] Institute for Biomedical Engineering, Department of Information Technology and Electrical Engineering, ETH Zurich, Switzerland

[3] The Roger Williams Institute of Hepatology, Foundation for Liver Research, London, UK

[4] Faculty of Life Sciences and Medicine, King's College London

[5] Department of Biomedical Sciences, University of Lausanne, Lausanne, Switzerland

[6] Department of Gastroenterology and Hepatology, University Hospital Zurich, Zurich, Switzerland

[7] Nuclear Physics Group and IPARCOS, Complutense University of Madrid, Madrid, Spain

[8] Health Research Institute of Hospital Clínico San Carlos (IdISSC), Madrid, Spain

[9] Inserm U1313, Université et CHU de Poitiers, Poitiers, France

[10] iThera Medical GmbH, Munich, Germany

* Email: daniel.razansky@uzh.ch

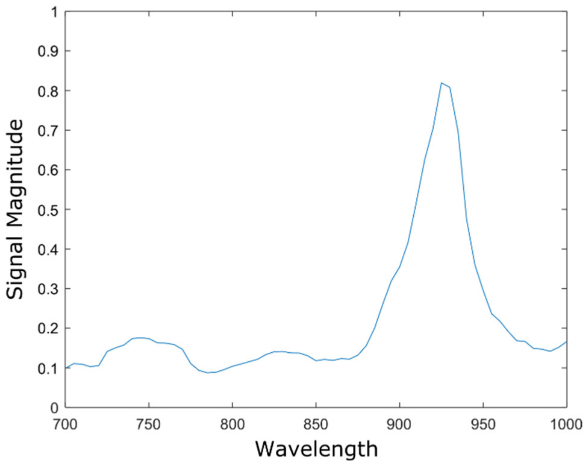

Figure S1: MSOT imaging of an excised fat tissue. Measured spectrum of excised fat tissue between 700 nm and 1000 nm excitation wavelengths.

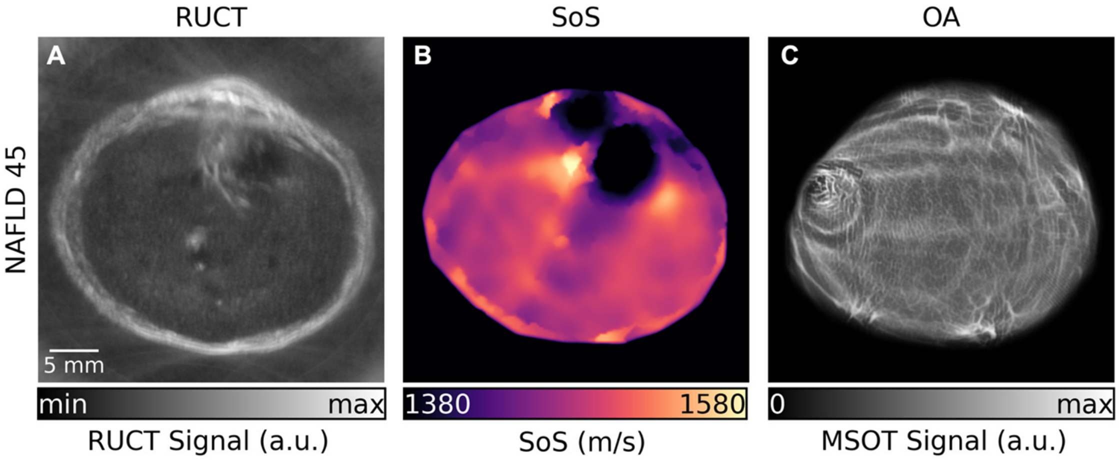

#

Figure S2: *In vivo* TROPUS images from 45 weeks old NAFLD mice. (A) RUCT image. **(B)** SoS image. **(C)** MSOT image. RUCT: reflection ultrasound computed tomography; SoS: speed of sound; MSOT: multispectral optoacoustic tomography.

#

#
